# Trends in turnover and turbulence at a large academic medical center before and during COVID-19: Analyzing structured clinical research professional roles

**DOI:** 10.1017/cts.2025.10063

**Published:** 2025-06-13

**Authors:** Marissa Stroo, Camila Reyes, Christine Deeter, Stephanie A. Freel, Heather Gaudaur, Richard Sloane, Denise C. Snyder

**Affiliations:** 1 Duke Office of Clinical Research, Duke University, Durham, NC, USA; 2 School of Medicine, Duke University, Durham, NC, USA; 3 Clinical and Translational Science Institute, Duke University, Durham, NC, USA; 4 Rewards and Recognition, Duke Human Resources, Duke University, Durham, NC, USA

**Keywords:** Clinical research workforce, clinical research professionals, employee turnover, workforce development, workforce turbulence

## Abstract

**Introduction:**

High workforce turbulence has plagued clinical research, becoming intensified during the COVID-19 pandemic, especially for patient-facing workers. In a time of great uncertainty and risk among healthcare workers, researchers included, the pandemic also brought increased demand for research studies in volume, speed, and complexity, triggering elevated staff turnover. This has posed significant hurdles for employers, especially research sites, where retaining skilled patient-facing clinical research professionals (CRPs) is pivotal for sustaining medical innovation. Lack of job standardization and advancement pathways has been noted to play an important role both in turnover and contributes to the inability to accurately measure workforce trends. To address these factors, Duke University adopted a competency-based job classification system for CRPs in 2016.

**Methods:**

Since that adoption of competency-based jobs, employee-level staffing data for all CRPs have been tracked monthly, creating a master data file from September 2016 through June 2024. This study updates previous analyses, evaluating turnover and turbulence rates, and demographic changes in the CRP workforce over this period.

**Results:**

Over the last six years, the Duke CRP workforce remained relatively stable. Voluntary turnover rates fluctuated, peaking at 19.1% in FY 2021 during the COVID-19 pandemic, and have steadily declined each year since then.

**Conclusions:**

Despite national workforce challenges exacerbated by the pandemic, our data indicate that proactive measures to standardize clinical research jobs and assess the resultant well-defined site-based employee data may have mitigated extremes in workforce turnover at Duke University. Turbulence rates, while stabilizing, signal areas for further study.

## Introduction

Retaining skilled clinical research professionals (CRPs) is an essential aspect of site quality [1–4]. The clinical research environment is characterized by rigorous regulations and increasing complexity, necessitating extensive training and meticulous oversight, particularly for employees newly entering the field or new to the site. Due to the time-sensitive nature of research studies, rapid participant enrollment and study completion are paramount. Consequently, turnover can prove to be highly detrimental, incurring substantial financial costs and lost productivity. The cost associated with replacing a clinical research coordinator has been approximated to range from $50,000 to $60,000 [5], which accounts for recruitment expenses and the time required for employee onboarding. This amount does not factor in the decreased study productivity during the interval, or the potential expenditures incurred due to the departure or burnout of other staff members compelled to assume additional responsibilities during that period [6].

The COVID-19 pandemic exacerbated already challenging staffing trends in the clinical research industry [7–10], in part by increasing the volume and demand for research studies and clinical trials. While, the “Great Resignation” has been touted as a characteristic of workforces everywhere beginning in 2021, Fuller and Kerr [11] argue this phenomenon was an extension of an already growing trend of resignation rates spanning more than a decade. The Society for Clinical Research Sites (SCRS) addressed the issue of increased turnover in a 2022 open letter to Sponsors and contract research organizations (CROs) addressing staff attrition. SCRS noted that reported rates of staff turnover increased from a range of 10%–37% pre-pandemic, to 35%–61% [12]. They noted the impact that this has had on enrollment and study productivity, as it can take sites time to hire and train staff members, especially those who are patient facing, also contributing to increased site costs [13]. Altogether, data show that the COVID-19 pandemic resulted in increased turnover across many fields, and especially within healthcare workers [14,15].

The SCRS letter [12] did not address turbulence (staff movement within the organization). Staff movement across the organization can create similar challenges to attrition and may lead to study slowdowns due to work redistribution and hiring. However, this movement can be managed to reduce study disruption. The overall impacts of turbulence have not been published similarly to turnover, yet it is worth assessing within an organization to identify trends. This movement can be driven by various factors, including reasons that are positive, neutral, or negative. Examples of positive factors include promotions and opportunities to explore new therapeutic areas. Neutral factors encompass natural project shifts or endings. Conversely, negative factors may arise from issues with colleagues, managers, or leadership, or may be a sign of inequitable reward and recognition practices across an organization [16]. Categorizing the precise reasons for employee movement can be challenging, but a comprehensive analysis of turbulence trends across the enterprise can serve as a warning system for potential issues.

Even when driven by positive factors, turbulence can significantly impact study performance. It may result in slower participant recruitment due to study holds and may lead to participant retention challenges [17]. Turbulence may necessitate the hiring of new personnel, which incurs associated costs. There are no standard rates available in the literature to serve as a comparison benchmark for the level of turbulence observed in this study. Nevertheless, recognizing the potential implications of turbulence on research outcomes, it becomes imperative to measure and understand its prevalence within the clinical research workforce over time.

Turnover and turbulence rates for CRPs at academic medical centers (AMCs) are understudied and lack comprehensive data [18,19]. Our institution previously evaluated and published data on turnover in our clinical research workforce as it related to the implementation of a competency-based job framework [20], which allows for a CRP job dataset that is unique for academic Institutions and other health center sites. That published analysis covered a period up to August 31, 2019. Months later the COVID-19 pandemic drastically impacted the clinical research landscape, leading to anecdotes of drastically increased turnover for CRP jobs. The aim of this manuscript is to share lessons learned from an extended evaluation of this unique dataset, exploring trends and rates of attrition and turbulence for CRP employees during the four-year period since our prior publication and over the course of the COVID-19 pandemic. The goals of our analysis were to better understand rates over time and to evaluate pandemic impacts on these roles at one large AMC.

## Materials and methods

In 2016, Duke mapped all CRPs into 12 laddered job classifications, moving all employees who work primarily within the JTFCTC competencies from a number of administrative and other research positions into discrete and well-defined clinical research staff roles [21]. With the career pathway now in place, it enables us to analyze and monitor data related to our CRP workforce. The Duke Office of Clinical Research, in collaboration with the Duke University Human Resources Management Center, extracts quarterly reports of employee-level data related to staff movement into, out of, and within any of our CRP classifications, including employees who work in our cancer research center. These reports are cleaned and combined to create a running master data file of all CRP movements since September 2016. In our prior publication [20], we evaluated data for 3 years pre- and post-implementation of these new job classifications (September 1, 2016 to August 31, 2019). Here we provide an updated analysis from that point forward through the COVID workforce crisis and to the end of the most recent fiscal year (FY) (June 30, 2024). One important caveat is that in our previous publication we presented data that did not directly align with the FY (June–July). We are including the data for FY 2018 and FY 2019, which covers July 1, 2017, to June 30, 2019, to allow for evaluation of longer-term trends across fiscal years but note that it does not line up directly with the results in the initial turnover publication due to this temporal shift.

Our methods for evaluating turnover have been previously described [20], including the detailed methodology for determining change in employment status. In brief, attrition (separation) was defined as an employee from one of the 12 CRP classifications, voluntarily or involuntarily leaving Duke. Voluntary reasons [22] include resignation (*n* = 909, 92.5% of voluntary turnover), retirement (*n* = 66, 6.7%), and death (*n* = 8, 0.8%). Involuntary reasons encompass funding end, poor performance (termination for progressive disciplinary actions, based on behaviors such as missing deadlines or producing low-quality work), and policy violations (actions or behaviors by employees that go against the established rules, such as intentional disclosure of PHI). These align with standard human resources definitions used by other institutions.

Population sizes were calculated for each fiscal year start (July 1) to end (June 30). Attrition rates used the formula:

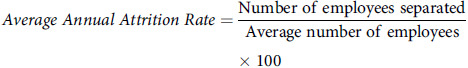




Age, tenure, sex, race, and ethnicity data from the HR reports were used to assess demographic changes in the workforce over time, as well as to create a snapshot of the workforce at the start and end of the evaluation period.

Turbulence was defined as employees moving organizationally within our CRP job classifications to a different research-based unit [known as Clinical Research Units (CRU)] within Duke. Job changes within the same CRU were excluded from analysis, as these often represented a promotion or a change in scope and may not have resulted in changes to participant facing activities or allowed for more flexibility in the transition period to prevent study disruption. Employees must complete a minimum of six months of successful service within a position before they are eligible for transfer or promotion, but current managers can provide exceptions to this rule if the circumstances support a more immediate change. Similar to the Attrition Rate, turbulence rates used the formula:

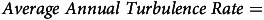




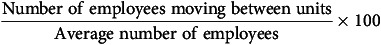




## Results

Data from a total of 2,169 unique Duke CRP employees were captured over the past 7 years. At the start of FY 2018, our workforce included 782 individuals, by the end of FY 2024 that number increased to 899 people.

As illustrated in Table 1, the CRP population has remained relatively stable over time. Each year includes all employees who were employed within any of the 12 CRP jobs at any time point during that fiscal year. The average age has trended slightly down over time, ranging from 44.9 to 39.8 years. The legal sex of the population remained predominantly female, with a notable increase from 85.5% in FY 2018 to 88.0% in FY 2023, dropping back down to 85.8% in FY 2024. Regarding racial diversity, the representation of Black, Asian, and individuals of two or more races showed variations, but remained relatively steady while the proportion of White CRPs decreased over the years. The data indicate a growing number of individuals who self-report unknown race categories. In terms of ethnicity, the majority of CRPs identified as non-Hispanic, with a slight increase in the percent who identify as Hispanic over the years. Efforts to increase the diversity of the workforce have increased over the past few years, and the resulting impact is likely not evident yet.

**Table 1.** Yearly clinical research professional population demographics (Fiscal year 2018–2024)

	FY 2018	FY 2019	FY 2020	FY 2021	FY 2022	FY 2023	FY 2024
**N**	936	1016	966	1014	1052	1021	1009
**Average Age**	44.9	40.5	40.5	39.8	39.9	40.0	40.2
**Sex (%)**							
Female	85.5	85.2	86.0	85.7	86.6	88.0	86.8
**Race (%)**							
White	70.2	70.5	71.5	69.1	67.4	66.8	63.8
Black	17.7	16.0	14.8	16.4	16.9	16.4	16.9
Asian	8.1	8.6	8.2	7.7	7.8	8.2	9.1
Two or more races	2.1	2.9	3.0	3.1	3.0	3.1	3.5
Other	0.6	0.6	0.5	0.4	0.8	0.7	0.6
Unknown	1.2	1.5	2.0	3.4	4.1	4.8	6.0
**Ethnicity (%)**							
non-Hispanic	95.3	93.3	93.8	93.6	93.6	92.9	92.4

FY = Fiscal year.

Over the period of FY 2018 to 2024, we observed the following trends in turnover and turbulence rates, with notable peaks in specific years (see Table 2 for details). Voluntary turnover has fluctuated, ranging from 14.6% to 19.1%. A peak in voluntary turnover was recorded in FY 2021 (July 2020–June 2021) at 19.1%. All turnover, encompassing both voluntary and involuntary departures, had been on a slight upward trajectory, starting at 16.1% in FY 2018 and reaching 19.4% in FY 2023, but dropped back to 16.9% in FY 2024. Turbulence, indicating movement across the workforce, has exhibited consistent levels over the years, with rates fluctuating between 3.1% and 6.2%.

**Table 2.** Yearly change comparison in attrition and turbulence rates (Fiscal year 2018–2024)

Fiscal Year	All Terminations (%)	Voluntary Terminations (%)	Turbulence (%)
**FY 2018**	16.1	15.1	6.2
**FY 2019**	18.9	17.0	3.7
**FY 2020**	16.3	14.6	4.6
**FY 2021**	20.1	19.1	4.8
**FY 2022**	19.4	17.9	4.6
**FY 2023**	19.4	17.5	4.4
**FY 2024**	16.9	15.5	3.1

**Figure 1.**
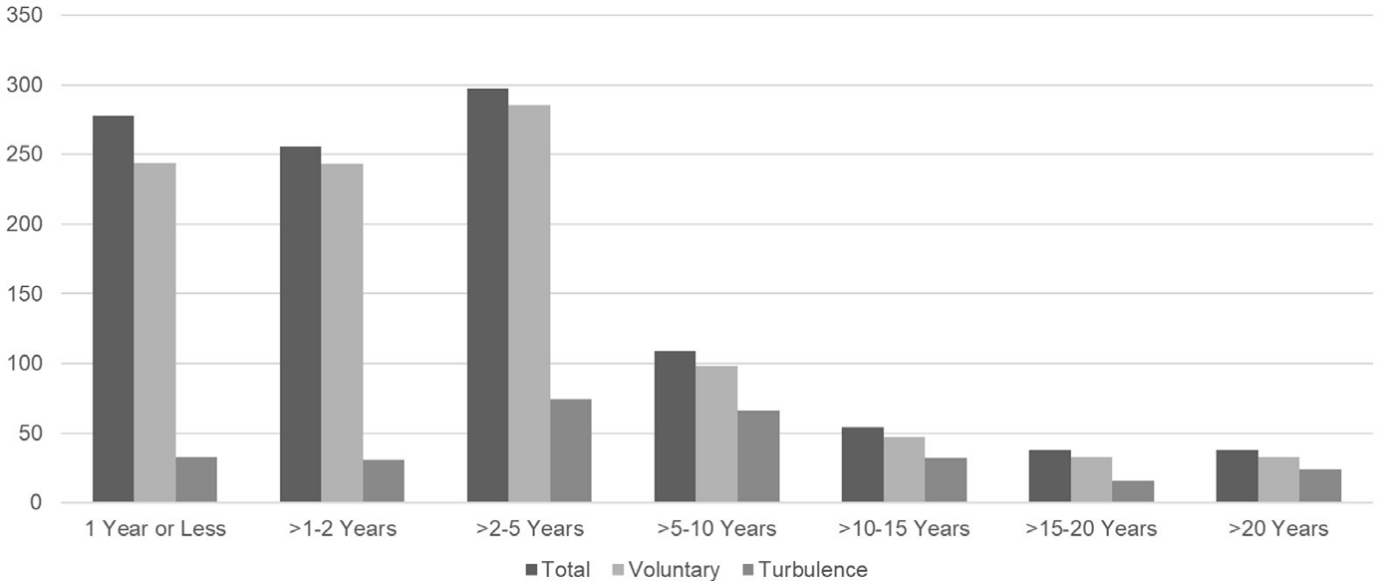
Turnover and turbulence rates by tenure (FY 2018–2024).

When we evaluated the data by tenure, a clear pattern is visible for turnover (see Figure 1). About a quarter of all turnovers happens within the first year of employment, another quarter within the second year, and almost 30% between years 3 and 5. In summary, 78.5% of all terminations and 78.9% of voluntary terminations occur within the first 5 years of employment. The termination rates for the remaining populations decrease over time. When we evaluate turbulence, we see that the rates peak between 2 and 10 years, with 24.2% (2–5 yrs) and 24.6% (> 5–10 yrs).

## Discussion

The COVID-19 pandemic had a profound impact on workforce stability across the globe. Remarkably, our data demonstrates a mitigation of this effect within our large AMC. We believe this mitigation is in part due to robust metrics tracking systems that enabled leadership from clinical research and HR to proactively and rapidly respond to and communicate with the well-defined CRP workforce as partners during periods of COVID-related instability. Additional efforts included a comprehensive market analysis in 2021, prompted by concerning global turnover trends that have been hailed as the “Great Resignation,” to which the clinical research industry was acutely susceptible. This market analysis resulted in broadly applied salary increases for CRPs at our institution in FY 2022. Over the FY 2018–2024 period, voluntary turnover exhibited an increase from 15.1% to 19.1% during the peak of COVID-related risk and instability but soon decreased to 17.9% and 17.5% in FY 2022 and FY 2023 respectively, as COVID vaccines and treatments became available and clinical research work returned to a more stable environment and pace and further decreased to 15.5% in FY 2024 mirroring rates prior to the pandemic.

Notably, in the years prior to our job mapping efforts, our average voluntary turnover rate was 20.3%, which was higher than any subsequent measure, even during the height of the pandemic. This lends further credence to our assertion that the benefits of standardized job classifications for the clinical research workforce positively impact retention and resilience of CRPs, as we described in our earlier manuscript [20]. It is also worth noting that the decrease we saw in turnover in FY 2024 is mirrored by the overall trends in the industry. An annual survey of research sites found that concerns regarding clinical research staffing had decreased from being reported as their largest challenge for clinical research sites (63% of sites reporting it as a challenge) in 2023, to being the fourth largest challenge (36% of sites) in 2024 [23]. No numbers are provided for rates of turnover in this survey, but the responses suggest that staffing challenges experienced during the pandemic are improving. Other reasons for lower turnover at our AMC may have included job security measures that were enacted to protect employees during “idle” time when studies were paused. This included matching staff to available work across the enterprise and transparent communication regarding COVID-19 impacts on clinical research from leadership in the form of town halls, training, and updates. This was all possible due to the well-defined CRP population and creation of a clinical research professional community. Our competency-based roles and collaborative HR partnerships facilitated agile, data-driven, responses during an unchartered time in navigating COVID-19.

Turbulence within the workforce, often overlooked and underreported, can similarly disrupt operations. While some turbulence signifies career and workforce development, excessive job-hopping within an unstructured system can be profoundly disruptive. At our AMC, turbulence was first measured in FY2018, and was at 6.2% then, and has since decreased, averaging 4.2% from FY 2019 to FY 2024, with the lowest level of 3.1% in the most recent FY 2024. The data from before the job mapping cannot be accurately evaluated for historical rates due to the large volume of potential job classifications employees could be in, and the fact that these were not exclusively used by clinical research.

It is not surprising to us that attrition is highest during the first 2 years of employment given the nature of early and entry level positions in academic research historically [7,13,24]. Employees often use CRP positions as stepping-stones in their educational and career journeys, particularly when they serve to help enhance an application for medical or graduate school. This turnover can be seen as a natural part of the career progression process and an important part of the academic mission, supported by AMCs. Outside of this use, CRP roles remain less well-known by many new graduates. This lack of awareness of the intricacies of clinical research jobs may contribute to higher early-career turnover rates as new employees discover what the jobs entail and assess alignment with their own career goals. While our analysis of attrition over the course of staff tenure is not surprising, the data provide valuable insights into employee tenure patterns, shining light on potential areas for retention and management strategies. For instance, high levels of early career attrition suggest that improved onboarding, continuing professional development opportunities, additional training for managers on how to support early career employees, and awareness campaigns for high school and college students may be areas for further exploration and intervention. Likewise peaks of turbulence during mid-career may suggest that individual development plans with attention to creating leadership opportunities for employees may help to retain and advance employees within their current organizational units. Furthermore, these data support needs for standardized onboarding and training that can be delivered as quickly as possible maximizing work from staff new to clinical research.

One notable strength of this study lies in its substantial sample size, drawn from human resource data within a large AMC. Although the study focuses on a single workplace, it examines the dynamics of a sizeable and well-defined clinical research workforce situated in a highly competitive clinical research environment. Anecdotal evidence, from collaborations across academic and industry professional networks, suggests that turnover rates in this specific region of North Carolina may exceed national averages due to the abundant clinical research positions available in academic, CROs, and industry sectors.

However, our study faces limitations, primarily the reliance on Human Resource data inherently lacks context into the reasons why employees leave. To address this concern, we implemented an optional exit survey in FY 2019 to gather information about why employees leave the organization or choose to change roles. In this survey employees can indicate any of the reasons that contributed to their decision to leave the organization or move to a new job within the organization. Consistently, the top three reasons employees provide for why they are leaving or changing jobs are “Career advancement” (58% of respondents endorsed this as a reason in FY 2024), “Lack of training/orientation” (37% endorsed in FY 2024), and the “Working relationship with their manager or supervisor” (32% endorsed in FY 2024). These are aligned with ongoing efforts being made within the organization to address turnover and employee satisfaction. However, our exit survey has limited uptake (only 28.6% of employees who leave or change jobs complete the survey), so generalizing those results beyond internal use for improvement is not yet feasible.

There is no quick, one-time fix to improve employee retention. By using all the available data we have access to, we can begin to understand any potential red flags and possible motivations for employees leaving our institution identifying areas for improvement. These early signals allow us to know when and where we should gather more workforce culture data, such as satisfaction surveys, to aid in retention strategies. To that end, we are also piloting the practice of stay interviews as a means for improving manager and employee partnerships toward individual employee retention. Current initiatives identified by these efforts include expanding training for managers and enhancing our onboarding processes [25].

While the COVID-19 pandemic brought significant challenges to staffing trends within the clinical research industry, we observed surprising resiliency in our own workforce. One of the key takeaways from our experience is the fundamental importance of identifying your workforce. Clearly defined roles and HR data facilitated the tracking of staffing trends and targeted communications. Likewise, partnerships with HR to gain access to regular data and to build strategies for tracking and trending turnover and turbulence is critical before any meaningful evaluation of retention interventions can be accomplished [26]. These data-driven strategies show promise for enhancing workforce resiliency and retaining these essential members of the clinical and translational enterprise.
